# Pulsed Radiation Therapy to Improve Systemic Control of Metastatic Cancer

**DOI:** 10.3389/fonc.2021.737425

**Published:** 2021-08-23

**Authors:** Kewen He, Hampartsoum B. Barsoumian, Duygu Sezen, Nahum Puebla-Osorio, Ethan Y. Hsu, Vivek Verma, Chike O. Abana, Dawei Chen, Roshal R. Patel, Meidi Gu, Maria Angelica Cortez, James W. Welsh

**Affiliations:** ^1^Department of Radiation Oncology, The University of Texas MD Anderson Cancer Center, Houston, TX, United States; ^2^Department of Radiation Oncology, Shandong Cancer Hospital Affiliated to Shandong University, Shandong Cancer Hospital and Institute, Shandong First Medical University and Shandong Academy of Medical Sciences, Jinan, China; ^3^Department of Radiation Oncology, Koç University School of Medicine, Istanbul, Turkey; ^4^Department of Radiation Oncology, Allegheny General Hospital, Pittsburgh, PA, United States; ^5^Albany Medical College, Albany, NY, United States; ^6^Department of Immunology, The University of Texas MD Anderson Cancer Center, Houston, TX, United States

**Keywords:** radiation therapy, immunotherapy, metastatic cancer, adaptive immunity, memory effect

## Abstract

Radiation therapy (RT) is emerging as an interventional modality in the cancer-immunity cycle, augmenting the activation of an adaptive immune response against tumors. RT, particularly in combination with immunotherapy, can enhance immune memory effects and shape the tumor-directed T-cell populations. However, a single cycle of RT delivered to a limited number of polymetastatic lesions is rarely sufficient to achieve systemic control. We hypothesize that several rounds of RT, akin to several rounds of immunotherapeutic drugs, is likely to provide greater clinical benefit to patients with metastatic disease. We propose that the repeated exposure to tumor antigens released by “pulsed-RT” (i.e., treating 2-4 tumor lesions with 3 irradiation cycles given one month apart) may amplify the adaptive immune response by expanding the tumor-specific T-cell receptor repertoire, the production of high-affinity tumor antibodies, and the generation of memory lymphocytes and thereby improve immune control of systemic disease.

## Introduction

Radiation therapy (RT), along with surgery and chemotherapy, are the three original pillars of cancer treatment. More than half of patients with newly diagnosed cancer receive RT as part of treatment (1). The clinical effectiveness of RT results not only from local control *via* induction of DNA damage at the treatment site but also from the ability to induce immune-mediated systemic antitumor effects that may control metastatic disease (2–4). RT is referred to as an *“in-situ* vaccine” owing to its ability to trigger antigen-specific, adaptive immunity (2). RT affects many of the steps in the cancer-immunity cycle, which begins with the release of cancer-associated neoantigens and proceeds through the activation of innate and adaptive immune responses, culminating in tumor killing (3). Although RT given with immune checkpoint blockade has led to unprecedented responses in some patients, these responses mostly remain rare and transient. In this article, we first discuss the biological rationale for RT-mediated “vaccination” to enhance systemic immune responses. We then outline current challenges in treating polymetastases with RT. Next, we propose the concept of “pulsed-RT,” which we define as three cycles of treatment given to 2-4 lesions per cycle, and describe the potential mechanisms that seem to support the effectiveness of pulsed-RT.

## RT Induces Systemic Antitumor Immune Responses

### Converting Tumors Into *In-Situ* Vaccines

RT is known to prompt the release of neoantigens and activate antigen-presenting cells (APCs). At 30 to 60 hours after irradiation with moderate doses (e.g., 10Gy), tumor cells undergo immunogenic cell death, which leads to release of tumor antigens (5). Irradiated tumors can express somatic mutations that generate neoepitopes, which can serve as targets of more robust immune responses (6, 7). One case report of a patient with non-small cell lung cancer (NSCLC) who experienced a complete response to RT and CTLA4 blockade demonstrated a rapid *in vivo* expansion of CD8^+^ T-cells that recognized a neoantigen encoded in a gene upregulated by RT (8). Thus, RT may increase the expression of immunogenic mutations in the irradiated tumor, which subsequently leads to the formation of neoantigens and activation of tumor specific CD8^+^ T-cells.

### Linking Innate and Adaptive Immunity by Priming T-Cells

During immunogenic cell death, radiation-damaged tumor cells subsequently activate APCs by translocating or releasing endogenous damage-associated molecular patterns (DAMPs) such as, calreticulin, ATP, and high-mobility-group-box-1 (HMGB1) that activates TLR4. These signals collectively lead to a potent inflammatory cytokine response that promotes DC maturation, upregulation of costimulatory signals that facilitate cross-priming of cytotoxic CD8^+^ T-lymphocytes, and upregulation of chemokine receptors (4). This cascade of events will eventually activate and attract effector adaptive immune cells to elicit antitumor functions. Moreover, RT-induced damage results in production of cytoplasmic DNA fragments, the presence of which activates the cGAS/STING pathway (9). Specifically, the damaged nuclei of tumor cells release double-stranded DNA fragments into the cytosol. The cytosolic protein cGAS converts those fragments into cGAMP, which activates the protein STING. This leads to the production of type-I interferons (IFNs), which promote the recruitment and activation of DCs and lead to adaptive immune activation (9–11).

### Reprogramming the Tumor Microenvironment

Tumors are known to use several mechanisms to evade immune surveillance, including downregulation of MHC-I expression, suppression of effector cytokines and chemokines, and production of inhibitory soluble factors such as TGF-β (4, 12). Poorly immunogenic tumors can also impair effector T-cells by expressing inhibitory checkpoint ligands, such as PD-L1, leading to exhausted/nonfunctional phenotype. In addition, since tumors lack the expression of costimulatory molecules (CD80, CD86, OX40L, 4-1BBL) amongst others, the T-cells become anergic and get eliminated. Moreover, inhibitory immune cells, such as M2 macrophages, myeloid-derived suppressor cells (MDSCs), regulatory T-cells (Tregs), and stromal fibroblasts, can also drive T-cell suppression and apoptosis (13–15). Optimized RT at the right dose and fractionation schedule, could promote an inflammatory response in tumors by inducing IFNs and chemokines that attract T-cells (16, 17). Low-dose RT (<2Gy), for example, leads to repolarization of tumor-associated macrophages from M2 to iNOS^+^ M1 cells (18), and promotes the infiltration of effector CD4^+^ T- and NK-cells, accompanied by reduction in TGF-β cytokine (19). The RT induction of chemokines such as CXCL9, CXCL10, CXCL11, and CXCL16 facilitates the homing of T-cells to tumors (18, 20–22). The upregulation of MHC-I molecules, Fas, and natural killer group 2 member D (NKG2D) on residual tumor cells in response to higher doses of RT allows recognition by incoming T-cells, with subsequent release of effector cytokines and killing of tumor targets (4).

### Synergistic Combination of RT and Immunotherapy

Given the ability of metastatic tumors to disguise and evade immunity by armoring themselves with suppressive cellular and physical barriers, especially by forming an inhibitory stroma, the abscopal effect remains quite rare in the clinic (12). This indicates that RT by itself is insufficient to activate and maintain antitumor immunity, thus there has been increased interest in combining RT with immunotherapy. Our group previously showed that a single cycle of SBRT (12Gy×3 fractions) combined with a costimulatory OX40 agonist produced abscopal responses in a murine model of PD1-resistant lung adenocarcinoma while RT or OX40 agonist alone did not (23). Additionally, immune checkpoint inhibitors such as anti-CTLA4 and anti-PD1/PD-L1 liberate T-cells from immunosuppression and reduce exhaustion (24–26). Alternative immunotherapies (TIM-3, LAG-3, and TIGIT) are also under investigation to overcome T-cell exhaustion (27). Clinical findings have provided some evidence of added benefits of combining RT and immunotherapy (28–33). For example, in a phase II single-arm study, patients with oligometastatic NSCLC given pembrolizumab after completing locally ablative RT to all known sites of disease showed a median PFS of 19.1 months, which exceeded the historical median of 6.6 months (*P*=0.005), with no reduction in quality of life (34). In another randomized trial, the combination of pembrolizumab and SBRT (to one metastatic site) in patients with oligometastatic NSCLC did not extend PFS or OS (29). These findings suggest collaboration between RT and immunotherapy, but the inconsistencies underscore the need to explore new treatment strategies, such as increasing the number of RT cycles or irradiating additional metastatic lesions.

## Clinical Challenges of Treating Polymetastatic Disease With RT

Currently, the most accepted clinical indication for curative-intent RT in metastatic cancer is for oligometastatic disease (having 1-5 metastatic lesions) (35). Several randomized trials support using consolidative SBRT for oligometastases. The first trial involved 49 NSCLC patients with three or fewer metastatic deposits remaining after first-line systemic therapy (36). Long-term follow-up (median 39 months) confirmed a durable PFS benefit from consolidative SBRT (14.2 *vs.* 4.4 months, *P*=0.022) as well as significantly longer OS (41.2 *vs.* 17.0 months, *P*=0.017). Another trial used upfront systemic therapy and allowed up to five lesions (37). The trial was stopped early after an interim analysis (29 patients) revealed a significant improvement in median PFS in SBRT group (9.7 *vs.* 3.5 months, *P*=0.01) at a median follow-up time of 10 months. Similarly, the SABR-COMET trial with 99 randomized patients allowed up to 5 metastatic lesions to be irradiated with SBRT (up to three in any single organ system). Despite experiencing grade-2 or greater adverse events (including three deaths), the SBRT group had a longer median OS (41 *vs.* 28 months, *P*=0.09) and longer median PFS (12 *vs.* 6 months, *P*=0.001) compared to the control palliative therapy group (38). The benefit of consolidative SBRT for patients with larger numbers of metastatic lesions (>5) remains unclear given the lack of adequate representation in the aforementioned trials (37–39). One concern is that increasing the number of sites to be treated with SBRT substantially increases the clinical workload, as distinct clinical RT plans are required for each lesion. Thus, the clinical workflow may restrict the number of metastatic sites that can be treated at one time. We propose to circumvent workflow-based restrictions by using pulsed-RT, in which 2-4 larger lesions are treated per cycle for three cycles. Moreover, irradiating multiple lesions (>5) simultaneously in one setting may add unwanted toxicity that may be avoided by pulsed-RT strategy. Emerging data suggests that pulsed-RT may have distinct biologic advantages when compared with single course treatment, as outlined below.

## How Might Pulsed-RT Achieve Systemic Control?

Similar to conventional vaccines that may require “booster cycles” to generate long-term memory, the concept of pulsed-RT, involving repeated RT cycles, may rely on generating tumor-associated antigens with each treatment cycle to build cellular and humoral memory. Together, given the systemic antitumor immune effects induced by RT, the limitations of using SBRT for widespread metastases (especially 5 or more lesions), and the knowledge gleaned from vaccine agents, we propose that pulsed-RT, in which 2-4 lesions are treated per cycle for three cycles, may improve systemic disease control. A recently published preclinical study using personalized ultra-fractionated stereotactic adaptive radiotherapy (PULSAR) in combination with α-PD-L1 demonstrated that spacing radiation fractions 10 days apart would achieve better tumor control and immunological memory in “cold” tumors in comparison to traditional daily fractions (40). Our cytokine data discussed below, comparing RT to pulsed-RT with or without anti-CTLA-4 checkpoint inhibitor, confirms the observed outcomes with repeated cycles of RT regarding immune stimulation and systemic activation.

### Activation of Innate and Adaptive Immunity by Pulsed-RT

**Figure 1:**
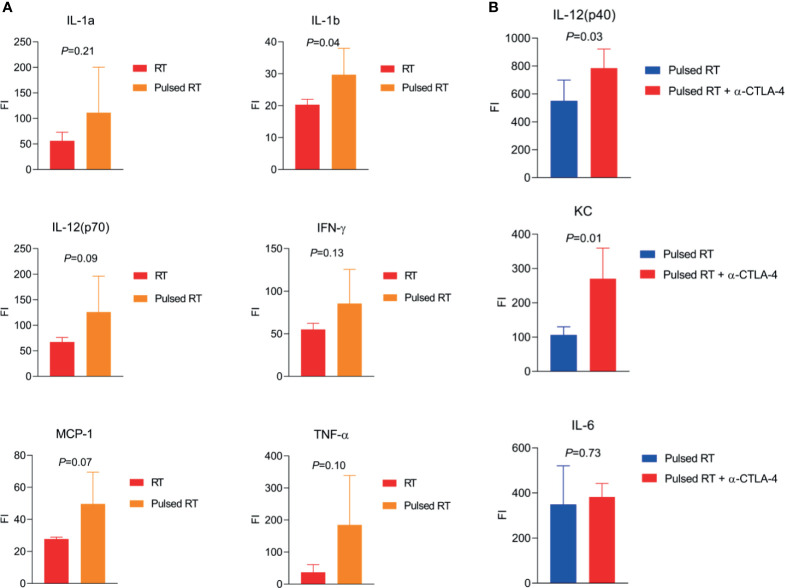
Effector cytokines produced by pulsed-RT, bridging innate and adaptive immunity. **(A, B)** The right hind legs of 129Sv/Ev mice were subcutaneously implanted with 2.5×10^5^ 344SQ-P cells to establish primary tumors, while the left legs were concurrently implanted with 1×10^5^ cells on day 0 to establish secondary tumors. Primary tumors were irradiated with 12Gy×2 on days 9 and 10, while secondary tumors were irradiated on days 15 and 16 post-tumor inoculation for the pulsed-RT group. Where applicable anti-CTLA4 was given i.p. on days 6 and 12. Sera were collected on day 19 (n=5 mice/group) and subjected to multiplex cytokine/chemokine analysis. Plates were read with Luminex platform and values were compared using Student’s t-tests.

To demonstrate that pulsed-RT could enhance macrophage and T-cell stimulation and effector functions, we have developed a bilaterally transplanted murine model of lung adenocarcinoma. The right hind legs of 129Sv/Ev mice were subcutaneously implanted with 2.5×10^5^ 344SQ-P cells to establish primary tumors, while the left legs were concurrently implanted with 1×10^5^ cells on day 0 to establish secondary tumors. Primary tumors were irradiated with 12Gy×2 on days 9 and 10, while secondary tumors were irradiated on days 15 and 16 with the same dose as primary. We also included control groups where only primary tumors were irradiated, and additional groups where anti-CTLA4 was given i.p. (50µg/injection). Sera were collected on day 19 post-tumor inoculation and analyzed by multiplex cytokines panel (BioRad #M60009RDPD). When comparing RT to pulsed-RT (**Figure 1A**), IL-1a (*P*=0.21) and IL-1b (*P*=0.04) proinflammatory cytokines were upregulated with pulsed-RT, which is indicative of innate immune-cell activation. IL-12(p70) cytokine was also upregulated (*P*=0.09) along with IFN-γ (*P*=0.13), which shifts the balance towards Th1 antitumor responses. Moreover, the monocyte chemoattractant protein-1 (MCP-1/CCL2) was increased (*P*=0.07), which usually helps attract monocytic precursors to the TME, that can further differentiate into M1 or M2 macrophages, therefore making MCP-1 act as a double-edged sword. At last, TNF-α cytokine was elevated with pulsed-RT treatment (*P*=0.10) which is previously shown by others to mediate abscopal responses (41) and favor M1 macrophage polarization (42). We next sought to investigate if using systemic anti-CTLA4 might augment the efficacy of pulsed-RT (**Figure 1B**). Indeed, IL-12 (40) cytokine (*P*=0.03) and KC (CXCL1) chemokine (*P*=0.01) were significantly upregulated, while there was no change detected in the Th2-polarazing IL-6 cytokine (*P*=0.73) (43).

### Pulsed-RT May Further Activate B and NK-Cells

**Figure 2:**
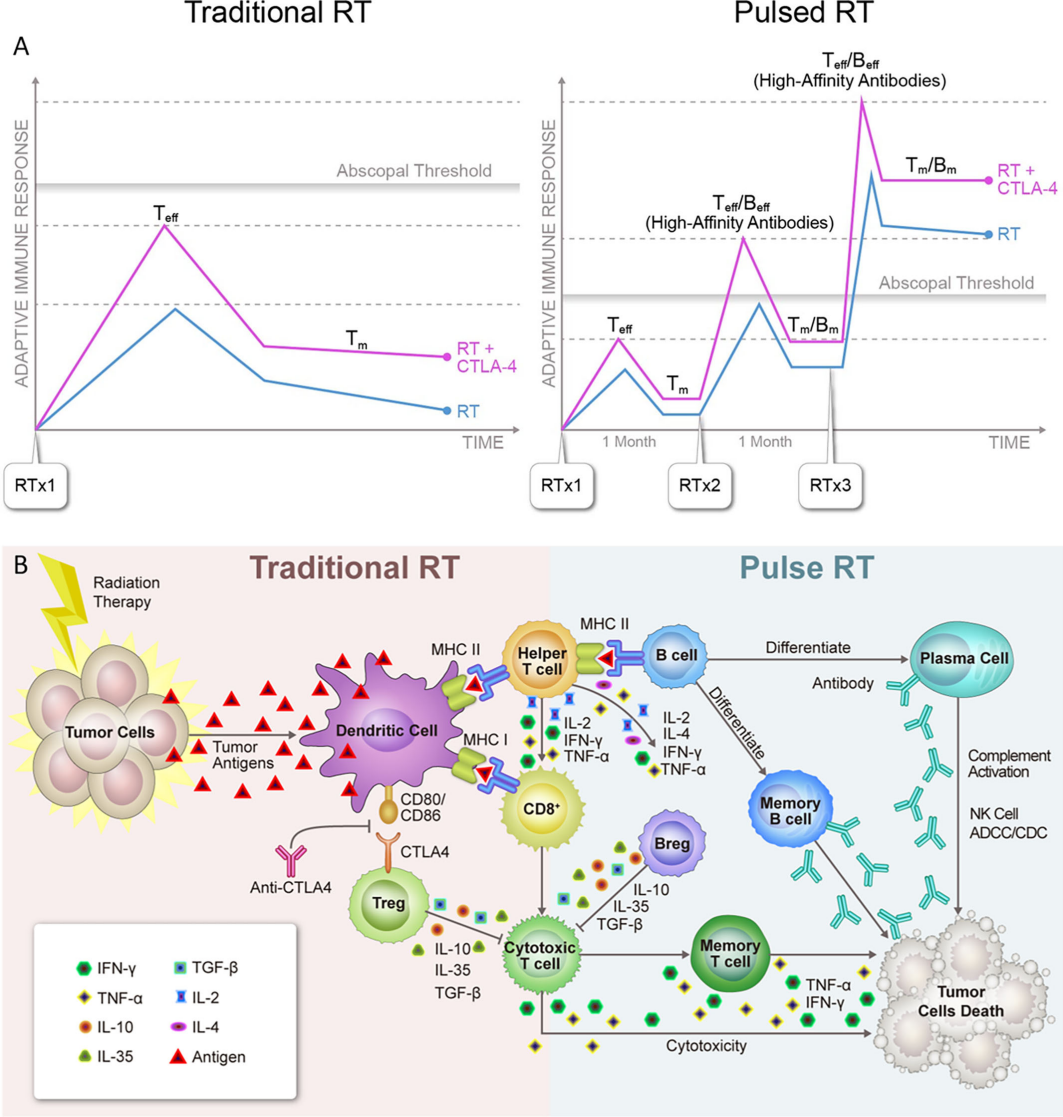
**(A)** Systemic effects of traditional *vs.* pulsed radiation therapy with anti-CTLA4. 1). Used with traditionally fractionated radiation therapy (RT), CTLA4 inhibitors boost the activation of T-cell clones with high-affinity T-cell receptors (TCRs) and expand the peripheral TCR repertoire, resulting in more robust activation of tumor-reactive T-cells and formation of memory T-cells (T_m_). However, this combination usually fails to reach the assumed abscopal threshold, which may explain why abscopal effects are so rarely observed in the clinic. 2). With pulsed-RT, the host immune system has a greater chance of being repeatedly exposed to the same tumor antigen, leading to immediate differentiation of memory cells into effector cells (T_eff_). Similarly, memory B-cells that differentiate into plasma cells would produce 10 to 100 times the number of antibodies than were produced during the primary response. As a result, the adaptive immune response triggered by pulsed-RT would presumably reach the abscopal threshold and have a greater chance of producing systemic response, which would be further improved by the concurrent use of anti-CTLA4. **(B)** Schematic overview of known and hypothesized functional interactions between lymphocytes in antitumor adaptive immune responses triggered by traditional vs. pulsed radiation therapy. 1). Traditional radiation therapy (RT) induces immunogenic cell death in cancer cells, releasing tumor neoantigens and activating antigen-presenting dendritic cells (DCs), which in turn migrate to local lymph nodes. In the lymph node, activated DCs present antigens to CD8^+^ T-cells through MHC-I molecules and CD4^+^ T-cells through MHC-II molecules. Clones of activated helper T-cells produce cytokines that initiate B-cells and CD8^+^ T-cells, which become cytotoxic T-cells. The latter ultimately leave the lymph node and travel to sites where cells bearing the target antigen reside, initiating a cytotoxic antitumor response. Conversely, at the time of the initial response to antigen, CTLA4 expressed on regulatory T-cells (Tregs) binds to CD80/CD86 on antigen-presenting cells (APCs) and inhibits T-cell activation. 2). In pulsed-RT, the initiated B-cells mature into effector B-cells and can further differentiate into plasma cells, which can produce antibodies against tumor-specific antigens. These antibodies in turn directly against their target proteins, triggering NK-cells−directed antibody-dependent cellular cytotoxicity (ADCC) or complement-dependent cytotoxicity (CDC) reactions. Effector B-cells can also enhance T-cell responses by producing stimulatory cytokines. As T and B-cells mature into effector cells, a subset of each differentiates into memory cells and can immediately become effector cells upon re-exposure to the same tumor-specific antigen. As a result, a secondary exposure to a given antigen would trigger an immune response that is much more rapid and more vigorous than that seen with the first pulsed-RT exposure. Conversely, regulatory B-cell (Bregs) can act in concert with Tregs to suppress antitumor immune responses. IFN, interferon; IL, interleukin; TGF, tumor growth factor; TNF, tumor necrosis factor.

RT-induced antitumor immune responses are predominantly attributed to MHC-I−restricted CD8^+^ cytotoxic T-cells, but MHC-II−restricted B- and NK-cells are also important drivers of antitumor immunity. Tumor-infiltrating B-cells are heterogeneous and have distinct functional subsets that can be either tumor-promoting or suppressing (44). For example, IL-10- and TGF-β-producing regulatory B-cells exert several protumor functions, while B-cells that are associated with tertiary lymphoid structures (TLSs), seem to be associated with more favorable clinical outcomes (44–47). TLSs are ectopic structures arising in inflamed, infected, or cancerous tissues that can generate an adaptive immune response (45–48). In one study of patients with melanoma, chemokine gene-expression signatures predicted the presence of TLSs and linked the presence of tumor TLSs with better OS (49). B-cells role can be summarized as 1) sampling local tumor antigens for presentation, 2) maturation to plasma cells and production of antibodies that mediate antibody-dependent cellular cytotoxicity (ADCC), 3) promote antitumor immunity by secreting IFN-γ and IL-12 as well as Granzyme B and TRAIL, which directly kill tumor cells (50). In the hypothesized process of pulsed-RT, a portion of effector B-cells differentiate into memory B-cells with each cycle (**Figure 2A**, blue curve), and can rapidly become effector cells again upon re-exposure to the same tumor-specific antigen. This translates into faster antibody production, facilitated ADCC through NK-cells engagement, and enhanced production of antitumor cytokines with subsequent RT cycle(s). Additional studies are warranted to further dissect how pulsed-RT may impact memory B-cell generation. On the other hand, NK-cells can also be activated *via* the NKG2D receptor, and RT has been shown to upregulate its ligand on various tumors (51, 52). Gathering the above, pulsed-RT treatment may enhance the crosstalk between B- and NK-cells and reprogram the TME in favor of antitumor outcomes.

### The RT−Immune Memory Effect

As noted previously, RT acts to prime and enhance the adaptive immune system. Pulsed-RT might enhance RT-immune memory effects, leading to the production of long-lived immune memory cells, which could be amplified by the addition of immunotherapy (**Figure 2A**). Tumor-rechallenge experiments in previous preclinical studies showed complete tumor rejection as well as increased antigen-specific memory CD8^+^ T-cells in mice that cleared initial tumors after combination therapy of RT and anti–PD-1/PD-L1 (26, 53). One supporting study with PULSAR radiation (given in two cycles, 10 days apart) showed better immunological memory upon MC38 tumors rechallenge, that was medicated by CD8^+^ T-cells (40). Moreover, the existence of an RT-immune memory effect is supported by a secondary analysis of KEYNOTE-001, suggesting that patients previously treated with RT had better response to anti-PD1 (pembrolizumab) given afterwards (54). Other immunotherapeutic agents, such as IL-2 with RT also seemed to produce synergistic immune-memory effects, especially in patients with a history of previous RT (median OS 8.8 *vs.* 7.34 months without previous RT, *P*=0.0116) (55). Although these findings from clinical studies are encouraging, the mechanisms underlying these observations have yet to be elucidated. One possibility is the presence of a T-cell subtype with tissue-resident memory (TRM), which is correlated with favorable prognosis in several types of cancer (56, 57). In one recent study, a proportion of T-cells showed increased motility and higher production of IFN-γ after RT and shared transcriptional-level similarities with TRM T-cells (58). These findings led the investigators to speculate that T-cells capable of surviving irradiation are more likely to have a TRM-like phenotype (58). With that rationale, pulsed-RT regimen may lead to the accumulation of more TRM T-cells with each cycle and prevent future tumor recurrence.

### Expanding and Diversifying Tumor-Specific T-Cell Receptor Repertoire With Pulsed-RT

Cancer vaccines aim to activate and stimulate the proliferation of tumor antigen-specific T-cells, however, this approach is limited by the fact that every patient’s tumor has a unique set of mutations. A melanoma study for patients who responded to epitope-loaded DC vaccine showed expansion of T-cells specific to the epitope, but also several other nonvaccine tumor antigens, suggesting that mobilization of a broad T-Cell Receptor (TCR) repertoire is required for tumor control (59). Preclinical work has demonstrated that RT-mediated tumoricidal effects largely hinge on both the presence and the specificity of cytotoxic T-cells (60). Addition of checkpoint inhibitors, such as anti-CTLA4 has been shown to augment RT to expand T-cell populations and increase diversity in TCR clonotypes (24, 53). For example, in a mouse model of triple-negative breast cancer showed that RT combined with anti-CTLA4 controlled both primary and metastatic tumors *via* tumor-infiltrating CD8^+^ T cells with a diverse TCR repertoire (61). Also, a recent clinical study revealed that a diverse and persistent TCR repertoire was associated with abscopal responses to RT and ipilimumab in patients with NSCLC (8). Similarly, our phase I-II clinical trial of SBRT plus ipilimumab in patients with metastatic tumors in lung or liver, partial response or stable disease (≥6 months) was associated with a higher number of peripheral CD8^+^ T-cells, higher CD8^+^/CD4^+^ ratios, and an increased number of either activated (4-1BB expressing) or potentially exhausted (PD1 expressing) CD8^+^ T-cells (62). RT has shown to induce the production of potential tumor antigens that leads to a phenomenon known as ‘epitope spreading’ (63–65), activating an array of T-cells, which in turn target the tumor to release another wave of antigens, creating a positive feedback loop (66, 67). These findings suggest that multiple rounds of RT (directed to a different lesion at each round) would increase the quantity and diversity of tumor-specific T-cells and further enhance this loop. Moreover, given the antigenic diverse nature of metastatic lesions, irradiating those tumors with a second or third pulse of RT will not only release tumor antigens shared with primary irradiated tumor, but will also release neoantigens specific to the metastatic site(s). The release of any identical antigens from the first and subsequent cycles of RT will contribute to the TCR repertoire clonality and the building of immunological memory in an incremental fashion (68), while releasing of neoantigens at metastatic sites will contribute to TCR repertoire diversity. Both clonality and diversity are important to prevent future recurrence and to generate systemic antitumor response.

## Future Directions With Pulsed-RT

When traditionally fractionated RT is used, the addition of CTLA4 inhibitors boosts the activation of T-cell clones with high-affinity TCRs and expands the peripheral TCR repertoire (69). However, RT alone or even with certain checkpoint inhibitors may fail to reach the assumed threshold for optimal induction of abscopal effects. When RT is delivered in pulses, the immune system is repeatedly exposed to the same tumor antigens, leading to faster deployment of memory T-cells. Similarly, memory B-cells that differentiate into plasma cells can produce 10-100 times more antibodies than those secreted during the primary immune response. Therefore, the adaptive immune response triggered by pulsed-RT could reach the abscopal threshold and have a greater chance of producing a systemic response that is further improved by concurrent use of anti-CTLA4 (**Figure 2A**). In conclusion, given the potential of pulsed-RT to generate more tumor-associated antigens, the pulsed-RT will act as an evolving “therapeutic vaccine” that improves systemic control. A schematic overview of known and hypothesized functional interactions between lymphocytes in antitumor adaptive immune responses triggered by traditional RT *vs.* pulsed-RT is illustrated in **Figure 2B**. Delivering RT in pulses may be especially beneficial for polymetastatic disease, in that it would provide a feasible and safe way to irradiate more metastatic lesions, while using current techniques and current clinical workflows. The optimal treatment schemes and potential mechanisms underlying this approach are worthy of further investigation.

## Data Availability Statement

The original contributions presented in the study are included in the article/supplementary material. Further inquiries can be directed to the corresponding author.

## Ethics Statement

The animal study was reviewed and approved by The University of Texas MD Anderson Cancer Center.

## Author Contributions

All authors contributed to the article and approved the submitted version. KH and HB provided figures.

## Funding

This work was supported in part by Cancer Center Support (Core) Grant P30 CA016672 from the National Cancer Institute, National Institutes of Health, to The University of Texas MD Anderson Cancer Center.

## Conflict of Interest

The authors declare that the research was conducted in the absence of any commercial or financial relationships that could be construed as a potential conflict of interest.

## Publisher’s Note

All claims expressed in this article are solely those of the authors and do not necessarily represent those of their affiliated organizations, or those of the publisher, the editors and the reviewers. Any product that may be evaluated in this article, or claim that may be made by its manufacturer, is not guaranteed or endorsed by the publisher.
